# Upregulation of Epithelial-To-Mesenchymal Transition Markers and P2X7 Receptors Is Associated to Increased Invasiveness Caused by P2X7 Receptor Stimulation in Human Glioblastoma Stem Cells

**DOI:** 10.3390/cells9010085

**Published:** 2019-12-29

**Authors:** Sihana Ziberi, Mariachiara Zuccarini, Marzia Carluccio, Patricia Giuliani, Lucia Ricci-Vitiani, Roberto Pallini, Francesco Caciagli, Patrizia Di Iorio, Renata Ciccarelli

**Affiliations:** 1 Department of Medical, Oral and Biotechnological Sciences, University of Chieti-Pescara, Via dei Vestini 29, 66100 Chieti, Italy; sihana.ziberi@unich.it (S.Z.); mariachiara.zuccarini@unich.it (M.Z.); marzia.carluccio@unich.it (M.C.); patricia.giuliani@unich.it (P.G.); patrizia.diiorio@unich.it (P.D.I.); 2 Center for Advanced Study and Technologies (CAST). University of Chieti-Pescara, Via L. Polacchi, 66100 Chieti, Italy; f.caciagli@unich.it; 3 StemTeCh Group, Via L. Polacchi, 66100 Chieti, Italy; 4 Department of Oncology and Molecular Medicine, Istituto Superiore di Sanità, Via Regina Elena 299, 00161 Rome, Italy; lriccivitiani@yahoo.it; 5 Institute of Neurosurgery, Università Cattolica del Sacro Cuore, Largo Agostino Gemelli 8, 00168 Rome, Italy; roberto.pallini@Unicatt.it

**Keywords:** glioblastoma stem cells (GSCs), epithelial-to-mesenchymal transition (EMT) markers, GSC invasiveness, transforming growth factor beta, BzATP, P2X7 receptor splice variants A and B

## Abstract

Glioblastoma (GBM) stem cells (GSCs), which contribute to GBM unfavorable prognosis, show high expression levels of ATP/P2X7 receptors (P2X7R). Here, we reported that cells exposure to 2’(3’)-*O*-(4-benzoylbenzoyl)-ATP (BzATP), a P2X7R agonist, up-regulated the expression of markers associated to epithelial-to-mesenchymal transition (EMT), a process likely contributing to GSC malignancy, and increased GSC migration/invasiveness like the known EMT inducer, Transforming Growth Factor β1 (TGFβ1). These effects were coupled to phosphorylation of SMAD2, a downstream effector in the TGFβ pathway, suggesting its involvement in P2X7R-mediated activity in GSCs. All BzATP effects, including a decrease in the caspase 3/7 activity in GSC medium, were mostly counteracted by the P2X7R antagonist A438079. Finally, BzATP increased the subunit expression of two main human P2X7R splice variants, the full-length P2X7A and the truncated P2X7B, lacking the carboxylic tail, which have different functional properties depending on their arrangement. Since up-regulation of A/B subunits might favor their assembly into a heterotrimeric P2X7R with great sensitivity towards agonists and cell energy support, this is in line with increased EMT markers expression, cell migration/invasion and GSC survival observed following P2X7R stimulation. As in GBM microenvironment extracellular ATP levels may activate P2X7R, our data suggest a P2X7R role in GBM recurrence/invasiveness.

## 1. Introduction

Glioblastoma (GBM) is the most common malignant primary brain tumor in adults, characterized by high invasiveness and recurrence [1]. Unfortunately, the current therapy including maximal safe surgical resection followed by radio- and chemo-therapy is largely ineffective so that the prognosis is poor and most patients die within the first two years. As in other malignant tumors, GBM aggressiveness seems due to the presence of a restrict population of stem-like cells inside the tumor mass, called GBM stem cells (GSCs), which are characterized by high proliferative potential, long life-span and resistance to antitumor agents [2]. GSCs can be isolated from tumor specimens and cultured in vitro in conditions favoring the growth of neural stem cells, which allow them to mimic the phenotype and genotype of primary tumors more closely than serum-cultured cell lines [3,4]. Moreover, upon injection into immunodeficient mice, they are able to recapitulate human tumor [5], thus representing a useful model to study the efficacy of old and new anti-tumor therapies.

GSC aggressiveness seems to also rely on epithelial-to-mesenchymal transition (EMT), an important biological process usually involved in embryogenesis, tissue repair and wound healing [6]. Although still debated in a neuro-epithelial context like GBM, in primary epithelial tumors EMT favors a more aggressive phenotype in cancer cells, with enhanced migration and invasiveness ability [6,7]. These cells also express a number of mesenchymal features including molecules such as Snails, Twist, Zinc finger E-box-binding homeobox (ZEB), vimentin and N-cadherin, which are considered specific EMT markers. More in detail, Snails, comprising Snail1 and Slug, also called Snail2, are transcriptional factors able to inhibit epithelial-related genes by binding to E-box DNA sequences through their carboxy-terminal zinc-finger domains [8]. Other EMT transcriptional factors are represented by ZEB, i.e., ZEB1 and ZEB2, whose molecular structure contains two zinc-finger domains that bind to E-boxes in DNA regulatory regions, contributing to inhibit epithelial genes while activating mesenchymal genes, including vimentin [9]. ZEB proteins may play a crucial role in tumor development, invasiveness and drug resistance [10]. Further EMT transcription factors are Twist1 and Twist2, which promote or repress several EMT related genes, thus inducing the expression of a mesenchymal phenotype in epithelial cells [11]. Another feature of EMT is the so-called “cadherin switch” in epithelial carcinomas, accounting for a loss in the expression of E-cadherin, a key protein in the formation of cell–cell junctions, and increased N-cadherin expression [7]. Finally, vimentin is a major constituent of the intermediate filament family of proteins, ubiquitously expressed in normal mesenchymal cells, being responsible of the maintenance of cell integrity and resistance against stress. An increased vimentin expression has been reported in various epithelial cancers [12]. The occurrence and activity of EMT markers have been also reported in GBM [7]. Thus, in human GBM cell lines, Snail1 silencing decreases cell proliferation, invasion and migration [13,14]. As well, ZEB1 contributes to GBM progression, acting as a pro-tumor factor, and its expression inversely correlates with survival of GBM patients [15]. Moreover, in GBM cell lines the overexpression of Twist1 enhances their migratory capacity and the cytoskeleton reorganization, two features supporting cell invasiveness once transplanted in mice brain [16]. As for cadherin, some data [17] showed that its switch from E- to N-cadherin is not essential to prove EMT in gliomas. Similarly, it has become a controversial issue also in epithelial tumors. In GBM, the role of N-cadherin is probably not associated with its increased expression, but with its differential distribution in cell membrane, which can alter the tumor cell capacity of adhesion and motility [7]. Finally, an increase in the vimentin expression has been observed also in GBM [18]. Altogether, findings from GBM and related cell lines support the involvement of EMT in GBM malignancy.

Among the factors contributing to the EMT onset and progression, a crucial role might be played by purines. [19]. ATP, adenosine and related compounds are ubiquitous substances present inside and released from virtually all types of cells, including tumor cells, and are involved in multiple physiological and pathological processes [20]. In particular, both ATP and adenosine are implicated in tumor growth and recurrence [21,22]. Noteworthy, in most tumors including GBM, the extracellular concentrations of ATP and adenosine are very high as compared to the microenvironment of normal cells [23]. In line with this evidence, we previously reported [24] that all four metabotropic receptors for adenosine were expressed in GSCs derived from GBM surgical specimens of three different patients, with a prevalence of the subtypes A3 for adenosine, and, among P2 receptors (P2R) for ATP, the ionotropic P2X7 receptor (P2X7R) showed the highest levels. Nine different P2X7R splice variants (P2X7A–J) of this receptor have been individuated so far, being the human main functional isoforms the canonical full-length monomer A and the truncated isoform B with a shorter amino acid sequence. The receptor resulting from P2X7A subunit assembly (P2X7AR) is equipped with a pore, while that formed by a P2X7B trimer (P2X7BR) is provided only with a functional cationic channel. ATP, the natural agonist of P2R, interacting at millimolar concentrations with the P2X7R, opens the pore—where present—that is permeable to molecules up to 900 Da, inducing plasma membrane permeabilization as well as apoptotic and necrotic events [25]. Accordingly, in the aforementioned paper [24], we observed a dose-dependent growth inhibition of GSCs when they were stimulated for two consecutive days by elevated concentrations of ATP or 2’[3’]-*O*-[4-benzoylbenzoyl]-ATP (BzATP), a rather selective P2X7R agonist. The same drugs, at the concentration of 500 μM, also potentiated the cytotoxicity of temozolomide, an agent currently used in GBM therapy. In contrast, lower ATP concentrations, likely closer to the extracellular ones found in GBM microenvironment, open only P2X7R cationic channels. Indeed, the P2X7BR variant, lacking the pore, is today regarded as a pro-cancerous receptor, able to increase tumor growth and invasiveness [26,27].

Based on these premises, we aimed our study at investigating, in GSCs, the influence of P2X7R activation on: i) EMT process, ii) cell migration/invasion, iii) expression of P2X7R, with a particular interest towards the two main splice variants A and B. To these purposes, we administered BzATP only once to the cultures at concentrations ranging from 50 up to 200 μM, which did not result to be cytotoxic to the cells (see the Results section). Of note, we compared BzATP effects with those caused by cell exposure to Transforming Growth Factor β1 (TGFβ1), known EMT inducer. Our findings showed that P2X7R activation upregulated EMT marker expression and increased cell invasiveness in the examined cells, also protecting them from apoptotic events. Interestingly, these effects were coupled to increased expression of the two P2X7R splice variants, the co-assembly of which into a hetero-trimeric receptor would likely support the pro-tumor effect consequent to GSC exposure to relatively low concentrations of the P2X7R agonist, BzATP.

## 2. Materials and Methods

### 2.1. Chemicals

Disposables for tissue culture were from Falcon (Steroglass, Perugia, Italy). Dulbecco’s Modified Eagle’s Medium/Nutrient Mixture F-12 Ham (DMEM/F-12) was purchased from Sigma-Aldrich S.p.A. (Milan, Italy) as well as penicillin/streptomycin, amphotericin B, 2′[3′]-*O*-[4-benzoyl benzoyl]adenosine-5′triphosphate tri[triethylammonium] salt (BzATP) and all the other chemicals, unless differently indicated. 3-[[5-[2,3-Dichlorophenyl]-1*H*-tetrazol-1-yl]methyl]pyridine hydro- chloride (A438079) and 3-[6-methyl-2-pyridinyl]-*N*-phenyl-4-[4-quinolinyl]-1*H*-pyrazole-1-carbo-thioamide (A8301) were ordered from Tocris Bioscience (Space Import, Milan, Italy); Transforming Growth Factor β (TGFβ) and human Epidermal (EGF) and Fibroblast (FGF) growth factors were purchased from PeproTech (SIAL, Rome, Italy).

### 2.2. Cell Cultures

We used GSCs obtained from three different patients (who provided written informed consent to the study according to research proposals approved by the Institutional Ethics Committee of Fondazione Policlinico Gemelli, UCSC (Prot. 4720/17) with a primary GBM, the molecular profile of which has previously been reported [24]. Here, cells maintained the same numeration used in the aforementioned paper [24], even though they are now officially registered with a number corresponding to the patient from whom the primary GBM was isolated and the GSCs derived. Thus, the nomenclature of GSCs #1 is invariant (deriving from the patient #1), whereas GSCs #2 and #3 correspond to cells #28 and #83 in the new nomenclature [28,29]. It has also to mention that these cells have been previously characterized for some important characteristics. They indeed showed high self-renewal, stemness marker expression and resistance to chemotherapy drugs when cultured in vitro, whereas, when injected in the brain of immune-compromised mice, they were able to reproduce a tumor identical to the human one as for antigen expression and histological tissue organization [30,31,32]. In this study, GSCs were grown using a standard protocol previously reported [30]. Briefly, cells isolated from tumor were cultured and expanded in DMEM/F12 medium without serum but supplemented with some mitogens (20 ng/mL of human recombinant EGF and 10 ng/mL of human recombinant FGF-basic). Under these conditions, cells formed classical neurospheres and were expanded. However, the pharmacological treatments were performed on cells grown as monolayer obtained pre-coating culture plates with Matrigel (Corning, SIAL) dissolved in culture medium and then seeding the cells that were fed with the usual culture medium containing also the growth factors above mentioned (dilution 1:200). In this condition, GSCs maintained spherogenic properties [30,31], but their use allowed a more precise quantification of cell survival in vitro. GSCs were used from passage 5 to 10 throughout the study. During this period, we found no significant modification in cell morphology or response to applied drugs. 

### 2.3. Experimental Protocol

We exposed GSCs to different concentrations of BzATP ranging from 50 to 200 μM. We compared BzATP effects with those induced by 5–10 ng/mL TGFβ1, a known inducer of the EMT process. Both TGFβ1 and BzATP were administered only once to the GSC cultures, whereas the evaluation of different functional/biochemical parameters was carried out at different time points as indicated in the Results section. In some experiments, cells were pre-treated with the antagonist of P2X7R (A438079, 10 μM) or TGFβ type I receptors (A8301, 0.5 μM), which were added to the cells 1 or 2h, respectively, prior to BzATP or TGFβ. 

### 2.4. Real Time PCR

RNA was extracted from cells by TRIZOL (Invitrogen, Thermo Fisher Scientific, Milan, Italy). and its amount was measured by spectrophotometry (Nanodrop 2000 spectrophotometer, Thermo Fisher Scientific). Additionally, RNA integrity was tested by 1.5% agarose gel electrophoresis in Tris Borate EDTA (TBE) (89 mM Tris, 89 mM boric acid, 20 mM EDTA, pH 8.0) and subsequent gel analysis by RED analyzer (Cell Biosciences, Santa Clara, CA, USA). All samples were amplified by Turbo DNA-free kit (Invitrogen, Thermo Fisher Scientific). Reverse transcription was performed using 1 µg of total RNA/sample and high Capacity cDNA Reverse Transcription kit (Applied Biosystems, Foster City, CA, USA), following the manufacturer’s instructions. The reaction mixture was loaded to the Gene Amp PCR system 9700 (Applied Biosystem) undergoing the cycle at 37 °C for 120 min. 

Real-Time PCR was carried out with the ABI Prism 7900 Sequence Detection System (Applied Biosystems). Expression of ZEB1, N-cadherin and Snail1 as well as of P2X7 A and B variants was evaluated at 0, 12, 24, and 48 h in GSCs untreated or exposed to drugs. Some forward and reverse primers were purchased from Integrated DNA Technologies (IDT, Leuven, Belgium) and are the following: ZEB1, forward 5′-CAAGGTGGCCATTCTGTTAT-3′ and reverse 5′-CTAGGCTGCTCAAGACTGTAG-3′; N-cadherin, forward 5′-CAACTTGCCAGAAAACTCC

AGG-3′ and reverse 5′-ATGAAACCGGGCTATCAGCTC-3′; Snail1, forward 5′-CGTTTTCCAGACCCTGGTTA-3′ and reverse 5′-TGACCTGTCTGCAAATGCTC-3′; GAPDH (glyceraldehyde 3-phosphate dehydrogenase), forward 5′-CATCACTGCCACCCAGAAG-3′ and reverse 5′-CAGTGAGCTTCCCGTTCAG-3′. The SYBR™ Green PCR Master Mix (Applied Biosystems) was used to perform the assay. The custom TaqMan Gene Expression Assays for P2X7A (forward 5′ AGATGCTGGAGAATGGAGTG *3′,* reverse *5′* TTCTCGTGGTGTAGTTGTGG *3′*) and P2X7B (forward 5′-CCCATCGAGGCAGTGGA-3′, reverse 5′-TTCTCGTGGTGTAGTTGTGG-3′) were designed according to [33], and the TaqMan Universal PCR Master Mix (Applied Biosystems) was used according to standard protocols. Gene expression levels were normalized (ΔCt) by using as endogenous control the house keeping GAPDH for EMT markers and the β2-microglobulin (B2M, Hs99999907_m1, Applied Biosystems, Foster City, CA, USA) for P2X7A and P2X7B splice variants. The results were analyzed for relative quantitation among groups using the comparative 2 ^ΔΔCt^ method [34]. 

### 2.5. Western Blot Analysis

Cells, harvested at 4 °C in lysis buffer containing a protease inhibitor cocktail (Sigma-Aldrich), were centrifuged at 14,000 rpm (10 min, 4 °C). Protein amount was measured by BioRad protein assay (Bio-Rad Laboratories, Milan, Italy). Samples (usually 60 μg), diluted in sodium dodecyl sulphate (SDS)-bromophenol blue buffer, were boiled (5 min) and separated on 10% SDS polyacrylamide gels. Proteins, once transferred on polyvinylidene fluoride membrane, were blocked with PBS/0.1 % Tween20/5 % nonfat milk (Bio-Rad Laboratories) for 2 h at 4 °C and then overnight incubated at 4 °C with primary antibodies [polyclonal rabbit anti-P2X7R (extracellular), dilution 1:200 (Alomone Labs, Jerusalem, Israel)]; [polyclonal rabbit anti-P2X7R (intracellular, C-terminus) dilution 1:300 (Sigma-Aldrich)], (monoclonal rabbit anti-vimentin (Cell Signaling, Euroclone, Pero, Italy), dilution 1:1000), (polyclonal rabbit anti-N-cadherin (Cell Signaling), dilution 1:1000), (monoclonal rabbit anti-ZEB1 (Cell Signaling), dilution 1:1000), (polyclonal rabbit anti-Twist1 (Cell Signaling), dilution 1:1000), (monoclonal anti-phosphoSMAD2, (Cell Signaling), dilution 1:1000). Subsequently, the membranes were exposed to goat anti-rabbit HPR-conjugated secondary antibody (final dilution 1:5000, incubation for 1h at, room temperature; Bethyl Laboratories Inc., Montgomery, TX, USA). Sample equal loading was determined by stripping and re-probing the blots with an anti-ß-actin antibody (dilution 1:1000, incubation overnight at 4 °C; Santa Cruz Biotechnologies, Heidelberg, Germany). Immunocomplexes were visualized by chemiluminescence (ECL) detection system (GE Healthcare Life Sciences, Milan, Italy) and quantified by densitometric analysis (ImageJ software; U.S. National Institutes of Health, Bethesda, MD, USA).

### 2.6. Lactate Dehydrogenase Activity

Lactate dehydrogenase (LDH) levels were assayed in GSC medium as an index of necrotic cell death since LDH is a cytoplasm enzyme that can be released following cell membrane injury. To this aim, cells were seeded (3 × 10^3^ cells/well) in 96-well plates and incubated with drugs following the usual protocol. At the indicated time points, cells were incubated (45 min, 37 °C, 5% CO_2_) with a specific lysis buffer, then the plates were centrifuged (250 g, 4 min). Subsequently, 50 μL of supernatant from each well were transferred to a new 96-well plate, to which 50 μL of substrate buffer (composition: 0.7 mM p-iodonitrotetrazolium violet, 50 mM L-lactic acid, 0.3 mM phenazine methoxysulfate, 0.4 mM NAD, and 0.2 M Tris-HCl pH 8.0) were added. The plate suitably blanket was incubated in the dark (30 min, room temperature). The reaction was blocked adding 50 μL/well of stop solution. The absorbance was spectrophotometrically measured at 490 nm. The results, expressed as a percentage of the total LDH released from positive controls, consisting of cells exposed to 25 μL of 10% Nonidet P-40 (NP-40), were calculated as follows: (supernatant absorbance value—white absorbance value)/(supernatant absorbance + lysate absorbance) × 100. All reagents were purchased from Promega Italia (Milan, Italy).

### 2.7. Caspase 3/7 Activity Assay

Apoptosis of GSCs, exposed or not to P2X7R agonist/antagonist, was evaluated by the Caspase-Glo Assay (Promega Italia), which contains a pro-luminescent peptide DEVD-aminoluciferin that can be cleaved by caspases 3/7 liberating aminoluciferin. A thermostable luciferase, included in the kit, generates a luminescent signal that is proportional to caspase-3/7 activity. Briefly, caspase-3/7 detection reagent was added at 1:1 ratio to the culture medium and cells were incubated at 25 °C for 1 h. The luminescent signal was revealed by a luminometer (Veritas™ Microplate Luminometer Turner Biosystems, Sunnyvale, CA, USA). The background value, measured in a sample containing growth medium and caspase-3/7 detection reagent but no cells, was subtracted from each measurement.

### 2.8. MTS Assay

Cell proliferation was assayed by 3-[4,5-dimethylthiazol-2-yl]-5-[3-carboxymethoxy phenyl]-2-[4-sulfophenyl]-2H-tetrazolium (MTS), using the CellTiter 96^®^ AQueous OneSolution Cell Proliferation Assay (Promega Italia). The absorbance was evaluated by a microtiter plate reader (Spectracount™, PerkinElmer Life, Waltham, MS, USA) at 490 nm.

### 2.9. Scratch Assay

Drug effects on GSC migration were evaluated by scratch assay. Briefly, cells were seeded in 6-well plates at 3 × 10^5^ cells/well and once they reached 80% confluence were pretreated with 5 µg/mL mitomycin-C (Sigma-Aldrich) at 37 °C with 5% CO_2_ for 3 h to arrest cell proliferation. Cell monolayer was scratched by a sterile 200-μL pipette tip and then washed, being the edge of the scratch smoothed with PBS. After pharmacological treatments performed according to the experimental protocol, GSCs were observed at a phase contrast microscope (Nikon Eclipse TS100) and images were acquired, using the Zoom Browser EX software for Windows 10 (Canon Italia), prior to (0 h) and at 6 and 24 h after the monolayers were scratched. The migration area was quantified by densitometric analysis (ImageJ software, 64-bit Java 1.8.0_112) and expressed as percentage of closing =/[A_0_ − A_n_]/A_0_ × 100, A_0_ and A_n_ representing the initial wound area (t = 0 h) and the residual wound area at the time of measurement (t = *n* h), respectively. 

### 2.10. Transwell Migration Assay

This assay measures cell chemotaxis and invasion through the extracellular matrix. We used filter membranes (EMD Millipore Corporation, Billerica, MA, USA) with 8 μm size pore suitable to test cell migration. Thirty/fifty microliters of Matrigel were plated on the top of the transwell membrane inserts placed in a 24well-plate, which were then transferred into a 37 °C incubator for 15–30 min allowing Matrigel to form a thin gel layer. Approximately 5 × 10^4^ cells were plated in the upper chamber and 700 μL of the desired attractant was added into the bottom of the lower chamber. More in detail, in a set of plates GSCs were incubated in the usual culture medium; in another set a high percentage of serum (10%), used as an attractant for cells, was added to the usual medium; further two sets of plates were incubated in the usual culture medium in the presence of TGFβ1 or BzATP. When present, the P2X7R antagonist A438079 or the antagonist of TGFβ receptors, A8301, were added 1 or 2 h prior to the other pharmacological treatments, respectively. After 24 h the inserts were removed from the plate and a cotton-tipped was used to eliminate cells that have not migrated trough the membrane. The membranes were fixed using cold methanol, stained with crystal violet 0.2% and then washed as many times as needed to remove dye excess. Subsequently, the cells on the membrane undersurface were counted under a light microscope (at an average of five semirandom non-overlapping fields at 200× magnification).

### 2.11. Statistical Analysis

The results are expressed as means ± standard error of mean (SEM) of at least three replicates. The significance has been calculated using one-way analysis of variance (ANOVA) followed by Dunnett’s post hoc test (GraphPad Prism 6.0, San Diego, CA, USA). Difference was considered to be statistically significant at a value of *p* < 0.05. 

## 3. Results

The experiments in this study, like in a previous one [24], were performed on GSCs isolated from GBM of three different patients obtaining comparable results. 

### 3.1. Influence of P2X7R Activation and TGFβ1 on the Expression of Selected EMT Markers in GSCs 

**Figure 1:**
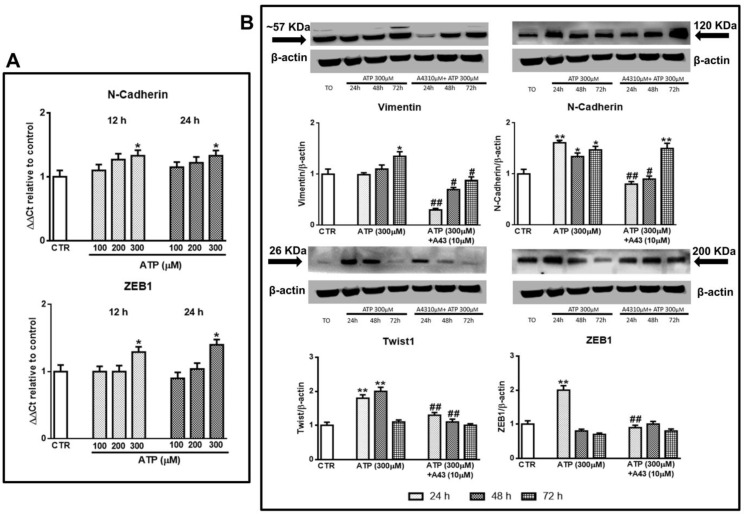
Effect of ATP on epithelial-to-mesenchymal transition (EMT) markers evaluated at different times after drug administration to cultured glioblastoma stem cells (GSCs). GSCs, cultured up to their confluence in vitro were exposed to different concentrations (**A**) or 300 μM of ATP (**B**), in the presence or not of the P2X7R antagonist, A438079, added to the cultures 1 h prior to ATP. (**A**) At the indicated time periods cells were collected and mRNA was extracted and analyzed for the gene expression of N-cadherin and ZEB1. mRNA levels were normalized (ΔCt) by using the house keeping GAPDH as endogenous control and the results were obtained by relative quantitation among groups using the comparative 2 ^ΔΔCt^ method. Values, calculated as fold of increase vs. untreated cells assumed as control (CTR) are the mean ± S.E.M. of three independent experiments where each sample was tested in duplicate. (**B**) cells, harvested at the indicated time periods, were lysed and the protein levels of EMT markers such as vimentin, N-cadherin, Twist1, and ZEB1 were determined by western blot analysis. Immunoblots were re-probed with an antibody against β actin, quantified by densitometric analysis, normalized to β actin used as an internal control, and reported in the histograms assuming the value of control/β-actin = 1. Immunobands in the figure are representative of independent experiments carried out in GSCs #1 and tested in triplicate. Of note, the band of β-actin is the same for all blots reported in the figures, which were obtained by cutting the same membrane at different heights corresponding to the molecular weights of the selected markers. *p < 0.05, **p < 0.01: statistical significance vs. untreated GSCs assumed as control (CTR); #*p* < 0.05, ##*p* < 0.01: statistical significance vs. cells exposed to ATP (one-way ANOVA plus Dunnett’s test).

We started our study performing pivotal experiments in which we exposed GSC cultures to ATP, the natural ligand for most subtypes of the purinergic P2R family. The selected ATP concentrations (100, 200, and 300 μM) were administered only once to the cultures and were lower than that (500 μM) able to cause a definite cytotoxicity to the cells [24]. In this condition, only the highest ATP concentration was able to increase the expression of some EMT markers, as evaluated by real time PCR (N-cadherin and ZEB1) at 12 and 24 h or by western blot analysis (N-cadherin, ZEB1 and also vimentin and Twist1) within 72 h. In particular, ATP enhanced the protein content of vimentin and N-cadherin up to 72 h, whereas the increase of Twist1 or ZEB1 proteins lasted 48 h or 24h, respectively (Figure 1A,B). Cell pretreatment with the P2X7R antagonist A438079 reduced ATP-induced effects, except that on N-cadherin at 72 h. 

**Figure 2:**
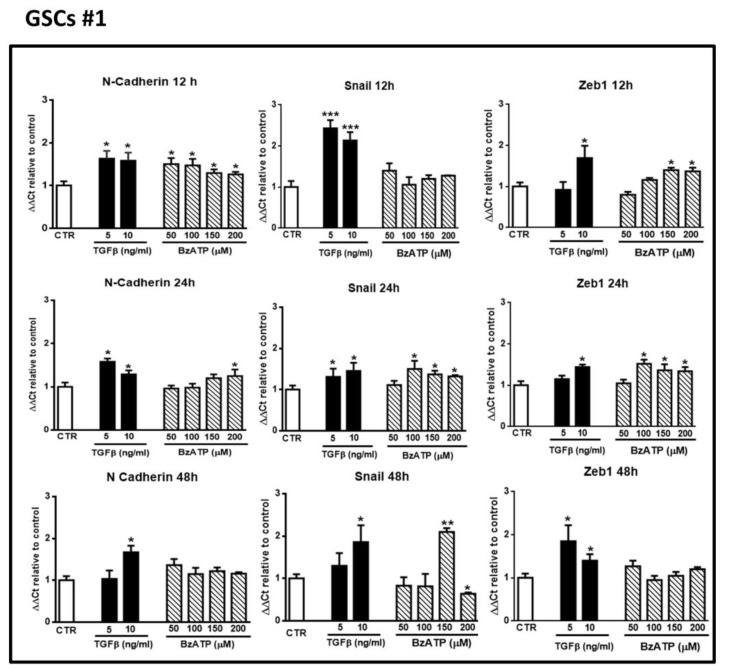
Effect of Transforming Growth Factor β1 (TGFβ1) or 2’(3’)-*O*-(4-benzoylbenzoyl)-ATP (BzATP) on EMT markers evaluated by qRT-PCR at different times after drug administration to cultured GSCs #1. GSCs, grown up to their confluence in vitro, were exposed to different concentrations of TGFβ1 or BzATP. At the indicated time points cells were collected to obtain their mRNA to analyze the gene expression of the indicated EMT markers. mRNA levels were normalized (ΔCt) by using the house keeping GAPDH as endogenous control and the results were analyzed for relative quantitation among groups using the comparative 2 ^ΔΔCt^ method. Values, calculated as fold of increase vs. untreated cells assumed as control (CTR) are the mean ± S.E.M. of three independent experiments, in which each sample was tested in duplicate. *p < 0.05, **p < 0.01, ***p < 0.001: statistical significance vs. CTR (one-way ANOVA plus Dunnett’s test).

**Figure 3:**
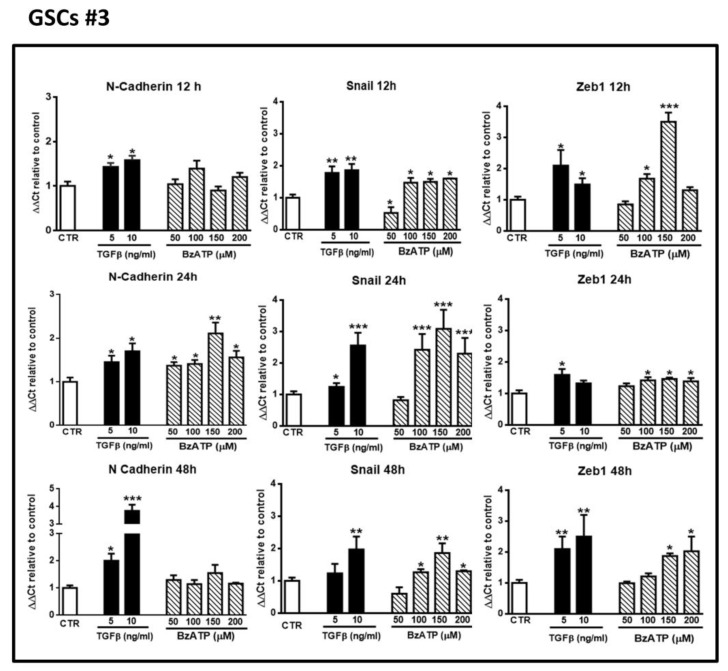
Effect of TGFβ1 or BzATP on EMT markers evaluated by qRT-PCR at different times after drug administration to cultured GSCs #3. Similar to what is reported in the legend of the Figure 2, confluent GSCs were exposed to TGFβ1 or BzATP for the indicated times, at the end of which cells were collected and their mRNA was analyzed for the gene expression of EMT markers. mRNA levels were normalized (ΔCt) by using the house keeping GAPDH as endogenous control and the results were analyzed for relative quantitation among groups using the comparative 2 ^ΔΔCt^ method. Values, calculated as fold of increase vs. untreated cells assumed as control (CTR) are the mean ± S.E.M. of three independent experiments in which each sample was tested in duplicate. *p < 0.05, **p < 0.01, ***p < 0.001: statistical significance vs. CTR (one-way ANOVA plus Dunnett’s test).

Next, we compared the activity of TGFβ1, a known EMT inducer, with that of BzATP, a rather selective P2X7R agonist, on the expression of EMT markers. As for ATP, both drugs were administered only once to the cultures. In GSCs #1 and #3, we observed that TGFβ1, yet at the dose of 5 ng/mL, enhanced mRNA levels of the selected EMT markers after 12–48 h of treatment. Similarly, cell exposure to BzATP caused an increase of all marker expression, although with some differences each other, related to the effect onset/duration (Figure 2 and Figure 3). Indeed, in GSCs #1 BzATP significantly enhanced mRNA levels of N-cadherin at 12 h, of ZEB1 at 12 and 24 h and of Snail1 mainly at 24 h, whereas GSCs #3 showed a greater responsiveness to BzATP, the effect of which was evident at 24 h for N-cadherin and up to 48 h for ZEB1 and Snail1. 

**Figure 4:**
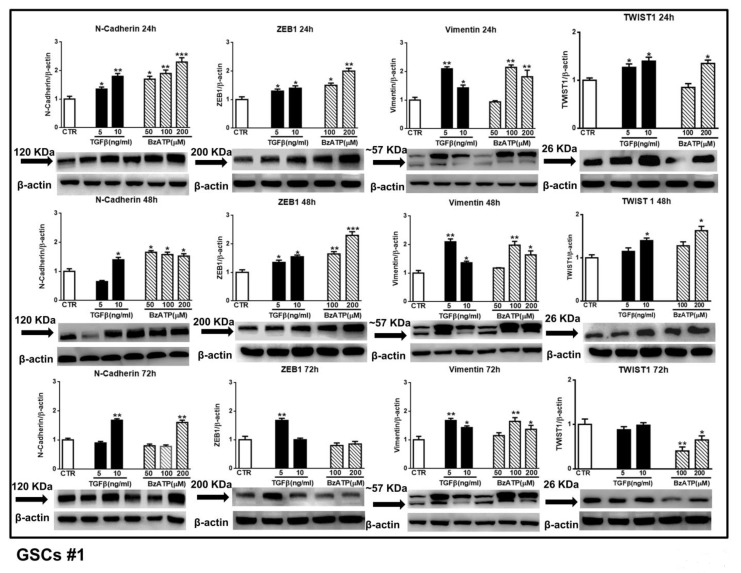
Effect of TGFβ1 or BzATP on EMT markers evaluated by western blot analysis at different times after drug administration to cultured GSCs. GSCs #1 were exposed to different concentrations of TGFβ1 or BzATP and harvested at the indicated times. The protein levels of EMT markers were determined by western blot analysis. Over the arrows close to the immune-bands the molecular weight (MW) of each EMT marker is reported according to the MW indicated in the data sheet of the used antibody. To note that for vimentin, which showed a double immune-band, as also reported in literature [35], the molecular weight is equal to about (~) 57 KDa to indicate an average of the weights of the two bands. Immunoblots were re-probed with an antibody against β actin, quantified by densitometric analysis, normalized to β actin used as an internal control, and reported in the histograms assuming the value of control/β-actin = 1. Immunobands in the figure are representative of three independent experiments. Of note, β-actin bands are the same for some blots reported in the figure, since they were obtained by cutting the same membrane at different heights corresponding to the molecular weights of the selected markers. *p < 0.05, **p < 0.01, ***p < 0.001: statistical significance vs. untreated GSCs assumed as control (CTR) (one-way ANOVA plus Dunnett’s test).

**Figure 5:**
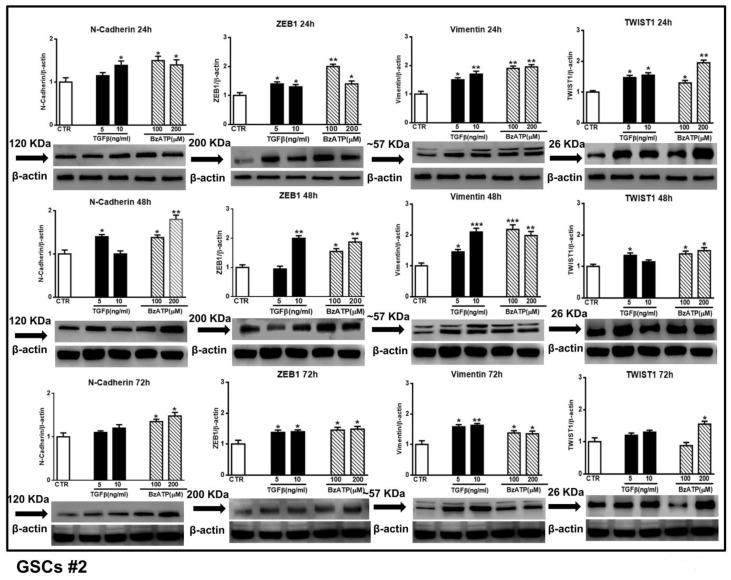
Effect of TGFβ1 or BzATP on EMT markers evaluated by western blot analysis at different times after drug administration to cultured GSCs #2. After exposure of the cells to different concentrations of TGFβ1 or BzATP, they were harvested at the indicated time periods. The protein levels of selected EMT markers were determined by western blot analysis. Immunoblots were obtained by exposing membranes to appropriate antibodies, recognizing proteins with different molecular weights (indicated close to the immunobands, over the arrows). Immunoblots were then re-probed with an antibody against β actin and quantified by densitometric analysis, the values of which, normalized to β actin used as an internal control, are reported in the histograms, assuming the value of control/β-actin=1. Immunobands reported in the figure are representative of three independent experiments using the same cells. Also in this case, the bands of β-actin are the same for the blots related to the same time point, as they were obtained by cutting the same membrane at different heights corresponding to the molecular weights of the selected markers. *p < 0.05, **p < 0.01, ***p < 0.001: statistical significance vs. untreated GSCs assumed as control (CTR) (one-way ANOVA plus Dunnett’s test).

These findings were corroborated by those obtained performing western blot analysis to evaluate N-cadherin, ZEB1, vimentin and Twist1 expression at different time periods. As shown in the Figure 4 and Figure 5, related to GSCs #1 and #2, both pharmacological agents were effective. In particular, in GSCs #1, BzATP caused a greater effect on N-cadherin and ZEB1 at 24 and 48 h after drug administration, thus reflecting the timeline of events observed by mRNA analysis. Moreover, the increase of vimentin was evident up to 72 h following cell exposure to TGFβ1 or BzATP whereas that of Twist was limited to the first 24 h. GSCs #2, like GSCs #3 (see data from real time PCR in Figure 3), showed a high responsiveness to BzATP, being the protein content of all EMT markers under investigation significantly increased throughout the 72 h observation period.

**Figure 6:**
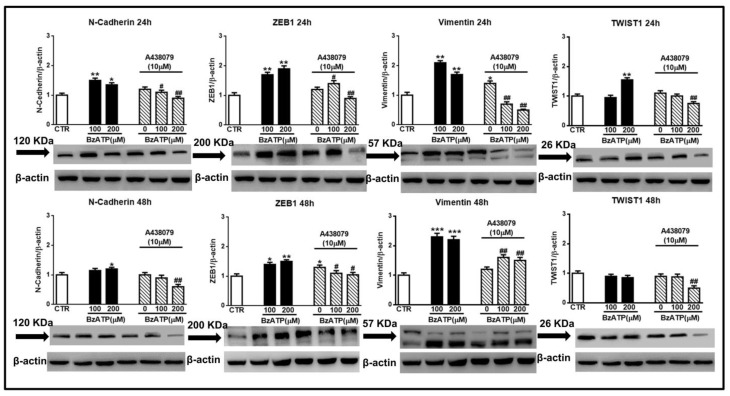
Blockade of the BzATP effect on EMT markers by the P2X7R selective antagonist A438079. Confluent GSCs were exposed to different concentrations of BzATP, alone or in the presence of the P2X7R antagonist, A438079 added 1 h before BzATP administration. Immunoblots were re-probed with an antibody against β actin, quantified by densitometric analysis, normalized to β actin used as an internal control, and reported in the histograms assuming the value of control/β-actin=1. β-actin bands are the same for some blots reported in the figure, since they were obtained by cutting the same membrane at different heights corresponding to the molecular weights of the selected markers. Immuno-bands reported in the figure are representative of three independent experiments carried out in GSCs #3. *p < 0.05, **p < 0.01, ***p < 0.001: statistical significance vs. untreated GSCs assumed as control (CTR); #*p* < 0.05, ##*p* < 0.01: statistical significance vs. GSCs treated with BzATP (one-way ANOVA plus Dunnett’s test).

Cell pre-treatment with the selective P2X7R antagonist A438079 substantially counteracted the effects of BzATP, mainly when this drug was administered at the concentration of 200 μM (Figure 6).

### 3.2. Effect of the Exposure of GSCs to the P2X 7R Agonist, BzATP, or TGFβ1 on Cell Migration and Invasion

**Figure 7:**
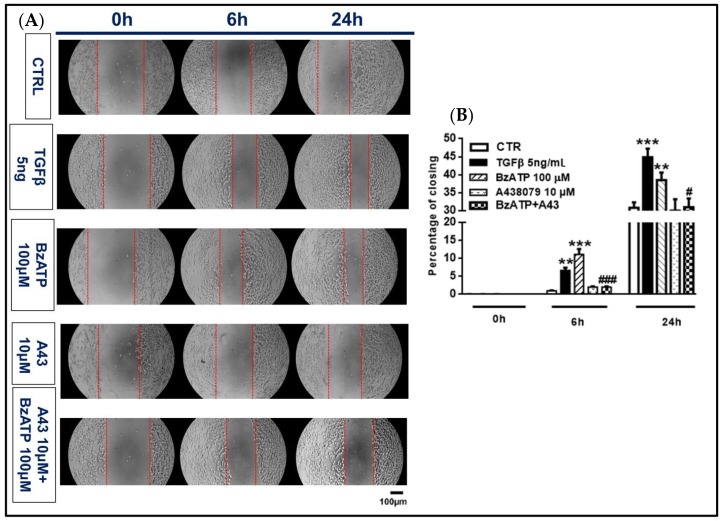
Effect of TGFβ1 and BzATP on GSC migration evaluated by scratch assay. Nearly confluent GSCs (80%) were incubated in the presence or absence of TGFβ1 or BzATP and when present, the P2X7R antagonist A438079 (A43), was added 1 h prior to the agonist. Cell were then scratch wounded and imaged at indicated times after injury. (**A**) Images of control and treated cells (GSCs #1) prior to (0 h) and 6 and 24 h after injury are representative of four independent experiments (scale bar = 100 μm for all panels) carried out for each cell type, giving similar results. (**B**) Quantification of the wound size was performed using ImageJ and graphed as percentage of wound closure. The values in the histograms are given as means± SEM and analyzed by one-way ANOVA and Dunnett’s post hoc test **p < 0.01; ***p < 0.001 vs. control (CTR); #*p* < 0.05, ###*p* < 0.001 vs. BzATP. Similar data were obtained using GSCs #2 and #3.

We then evaluated the effect of the treatments with TGFβ1 or BzATP on GSC migration and invasion. The scratch assay showed that both TGFβ1 and BzATP shortened wound healing at 6 and 24 h of treatment (Figure 7). 

**Figure 8:**
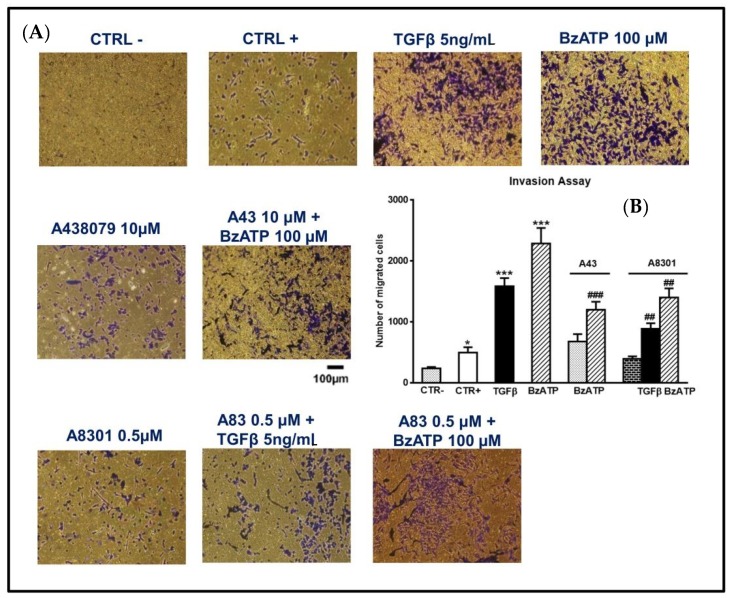
Effect of TGFβ1 and BzATP on GSC invasiveness evaluated by transwell migration assay. (**A**) GSCs, plated in the upper surface of a membrane inserted into a suitable plate, were incubated in the usual culture medium alone (CTR-) or in the presence of TGFβ1 or BzATP. In some experiments cells were pretreated with A438079 (A43), a P2X7R antagonist, or A8301 (A83), inhibitor of TGFβ type I receptors, which were added alone and 1 or 2 h, respectively, prior to the further pharmacological treatments. In another set of cells, a high percentage of serum (10%), usually acting as an attractant for cells, was added to the medium and cells were indicated as CTR+. The cells were allowed to migrate to the lower chamber for 24 h. The membranes were then removed and the cells, fixed and stained using crystal violet, were counted under a light microscope (at an average of five semirandom non-overlapping fields at 10x magnification). The images reported in the figure (scale bar = 100 μm) are related to GSCs #2 and are representative of three independent experiments carried out for each cell type, giving similar results. (**B**) Accordingly, values in the histograms are the mean ± SEM of these independent experiments and have been analyzed by one-way ANOVA and Dunnett’s post hoc test *p < 0.05, ***p < 0.001 vs. control (CTR+); ##*p* < 0.01; ###*p* < 0.001 vs. cells treated with TGFβ1 or BzATP.

Also, the transwell migration assay used to assess tumor cell invasiveness, demonstrated that both drugs enhanced cell migration through the membranes (Figure 8). 

In both types of experiments, the P2X7R antagonist A438079 abolished the BzATP-induced effect (Figure 7 and Figure 8). 

### 3.3. The Activity of BzATP on EMT Markers and GSC Migration is Linked to the Phosphorylation of SMAD2, A Downstream Effector of TGFβ Signaling 

**Figure 9:**
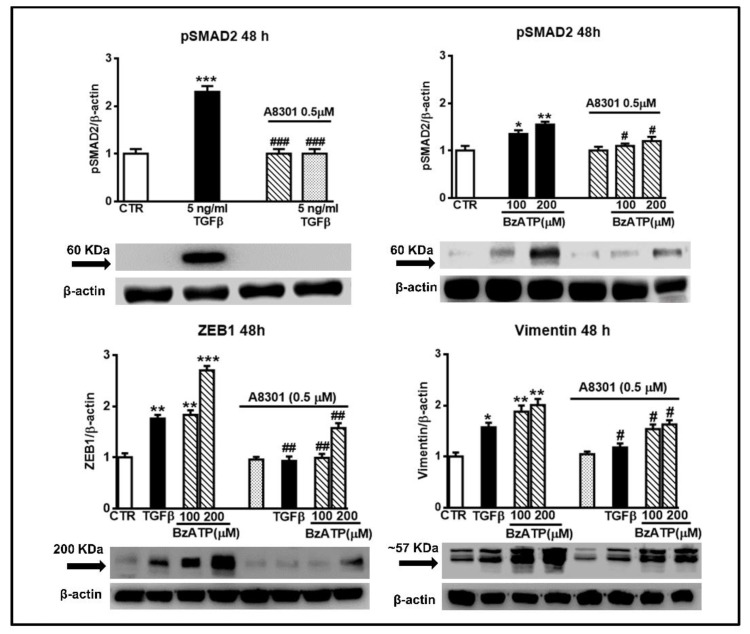
Increase in SMAD2 phosphorylation is involved in the effects promoted by either TGFβ or BzATP on EMT markers. GSCs were exposed to TGFβ1 or BzATP at the indicated concentrations. In some experiments, cells were pretreated with A8301, inhibitor of TGFβ type I receptors, which was added alone or 2 h prior to the other two drugs. After 48 h, cells were harvested and the protein levels of phosphorylated SMAD2 (pSMAD2) and some EMT markers were determined by western blot analysis. As for pSMAD2, different aliquots of proteins, derived from TGFβ or BzATP treated cells (60 or 80 μg, respectively), were used for the electrophoretic run, to make more evident the BzATP effect. Immunoblots were obtained by exposing membranes to the appropriate antibodies. Then, they were reprobed with an antibody against β actin and quantified by densitometric analysis, the values of which, normalized to β actin used as an internal control, are reported in the histograms, assuming the value of control/β-actin =1. Immunobands in the figure are related to GSCs #2 and are representative of three independent experiments carried out for each cell type giving similar results. *p < 0.05, **p < 0.01, ***p < 0.001: statistical significance vs. untreated GSCs assumed as control (CTR) (one-way ANOVA plus Dunnett’s test); #*p* < 0.05; ##*p* < 0.01; ###*p* < 0.001 vs. cells treated with TGFβ1 or BzATP.

As reported in glioma cell lines, TGFβ is an inducer of ZEB1-dependent mesenchymal trans-differentiation coupled to enhancement of their invasion capability. Interestingly, this effect was mediated by phosphorylation of SMAD2 [36], a protein involved in direct signaling of TGFβ type I receptors [37]. Since in our GSCs derived from patients’ tumor TGFβ had a behavior similar to that shown in GBM cell lines, i.e., as for ZEB1 and invasion increase, we wondered whether the effects caused by TGFβ and also by BzATP in our GSCs could be mediated by the same signal transduction pathway. Figure 9 shows that not only TGFβ but also BzATP, although to a lesser extent, increased SMAD2 phosphorylation. These effects were evident already after 24 h (data not shown), becoming maximal at 48 h. Accordingly, cell pretreatment with A8301, a potent blocker of the TGFβ type I receptors [36] was effective in inhibiting the TGFβ- and BzATP-induced increase in SMAD2 phosphorylation as well as the activity of both drugs on ZEB1 and vimentin expression (Figure 9) and GSC invasiveness (Figure 8).

### 3.4. Influence of BzATP on GSC Viability

**Figure 10:**
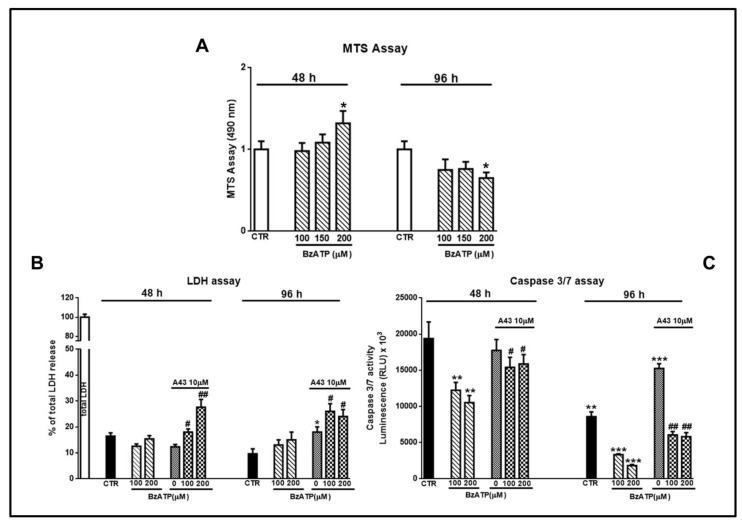
Evaluation of cell viability and of necrotic or apoptotic death induced by GSC exposure to BzATP. GSCs were treated with BzATP and in some experiments they were pretreated with the P2X7 receptor (P2X7R) antagonist A438079, administered 1 h prior to the agonist. After different times from the beginning of the pharmacological treatments cell viability (**A**), cell necrosis or apoptosis (**B**,**C**) respectively were assayed. (**A**) Cell medium was removed at the end of each time period and 3-[4,5-dimethylthiazol-2-yl]-5-[3-carboxymethoxy phenyl]-2-[4-sulfophenyl]-2H-tetrazolium (MTS) assay was performed. The values obtained by optical density at 490 nm for each sample are reported in the histograms, assuming the value of control = 1. (**B**) LDH release from cells, assumed as an index of necrotic death, was measured as reported in the Methods section. Values are expressed as the percentage of the total amount of the enzyme released in the medium from the cells after their lysis. (**C**) Apoptic death was assessed by the evaluation of the release of caspase 3 and 7, the most involved in this process, by luminescence using a commercial kit and following the manufacturer’s instruction. All values in the histograms are related to GSCs #3, but similar findings were obtained using cells #1 or #2. They are expressed as the mean ± S.E.M. of four independent experiments in which each sample was tested in triplicate. *p < 0.05, **p < 0.01, ***p < 0.001: statistical significance vs. untreated cells; #*p* < 0.05, ##*p* < 0.01: statistical significance vs. cells treated with BzATP (one-way ANOVA plus Dunnett’s test).

Finally, by MTS assay we found that cell exposure to BzATP at the chosen concentrations did not affect cell viability after 48 h, while modestly reducing it at 96 h, but only when used at 200 μM (Figure 10A). However, the evaluation of LDH and caspase 3/7 activity in the GSC culture medium, assumed as signals of necrotic and apoptotic death, respectively, showed that BzATP reduced the activity of the caspases without influencing that of LDH (Figure 10B,C). The effect of BzATP was once again counteracted by cell pre-treatment with the P2X7R antagonist that, when administered alone, increased LDH and caspases activity 96 h after its addition to the cells.

### 3.5. Expression of the Splice Variants P2X7A and P2X7B in GSCs in Basal Conditions and after Exposure to BzATP

**Figure 11:**
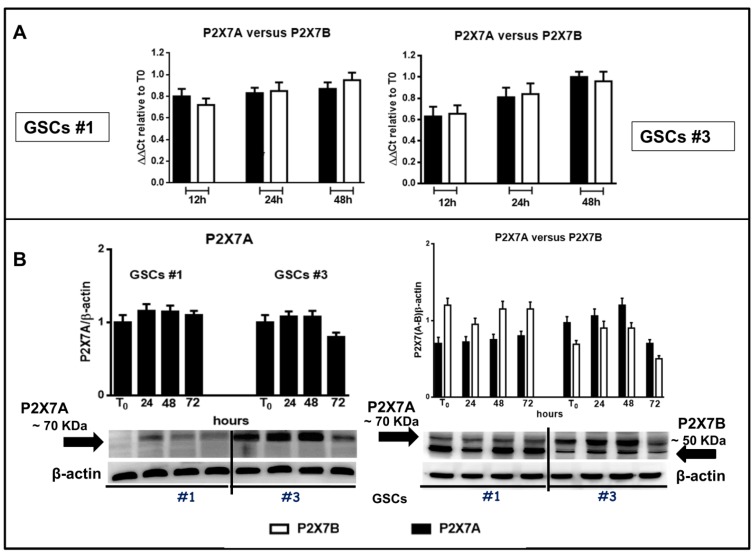
Expression of the two main human splice variants P2X7R A and B in GSCs. Confluent GSCs were harvested at different time periods to obtain their mRNA or proteins. (**A**) by qRT-PCR the evaluation of the mRNA levels was performed in relation to the principal P2X7R splice variants in humans that are the full-length form P2X7A and the truncated form (lacking the carboxy-terminal tail) P2X7B in GSCs #1 and #3 (panels on left or right, respectively). mRNA levels were normalized by using the house keeping β2-microglobulin as endogenous control. Values, calculated as fold of increase vs. those measured in cells at the beginning of the experiments (time 0) are the mean ± S.E.M. of 3 independent experiments performed using the cells above mentioned. Each sample was tested in duplicate. (**B**) protein levels of P2X7A and P2X7B monomers were determined by western blot analysis. Immunoblots were obtained by exposing membranes to two polyclonal antibodies, one recognizing among the others proteins at about 70 KDa and the second one recognizing proteins at about 70 and 50 KDa, respectively. Subsequently, immunoblots were re-probed with an antibody against β actin, to verify equal sample loading, and quantified by densitometric analysis. These values were then normalized to β actin and reported as such as for the histograms related to blots in which both P2X7R A and B subunits were recognized, whereas for the blots related to the only P2X7AR subunit the value were calculated assuming the ratio (cells at T_0_)/β-actin = 1. All values are the mean ± SEM of three independent experiments for each cell type.

Based on the evidence previously reported about the high expression levels of P2X7R in GSCs [24], we wanted to better define the role played by this receptor in GSCs by examining the expression of the P2X7AR and P2X7BR splice variants, which could influence GSC aggressiveness in different ways. By real time PCR and western blot analysis, we observed that both P2X7R isoforms are present in GSCs (Figure 11) and that the expression of the subunits of the full length P2X7AR and of the shorter P2X7BR was nearly constant during 48 h (Figure 11A). Similar results were obtained by western blot analysis (Figure 11B). 

In order to assess this aspect, we used two different antibodies, one directed against an extracellular loop common to both subunits forming the splice variants P2X7AR or P2X7BR, and another one directed towards the C-terminal tail, present only in the P2X7AR variant. This second antibody, more specific towards the P2X7AR subunit, was used to confirm data obtained with the first one. However, both antibodies are polyclonal; therefore, they identified more than one immune-band. Among them, both antibodies recognized a band at about 70 KDa, likely corresponding to the calculated molecular weight of the P2X7AR subunit, whereas the antibody against the extracellular loop also recognized, among the others, a protein at about 50 KDa, compatible with the molecular weight of the shorter splice variant P2X7BR (Figure 11B). Thus, western blot analysis would confirm the presence of proteins related to the two splice variants A and B of the P2X7R in the examined cells. 

**Figure 12:**
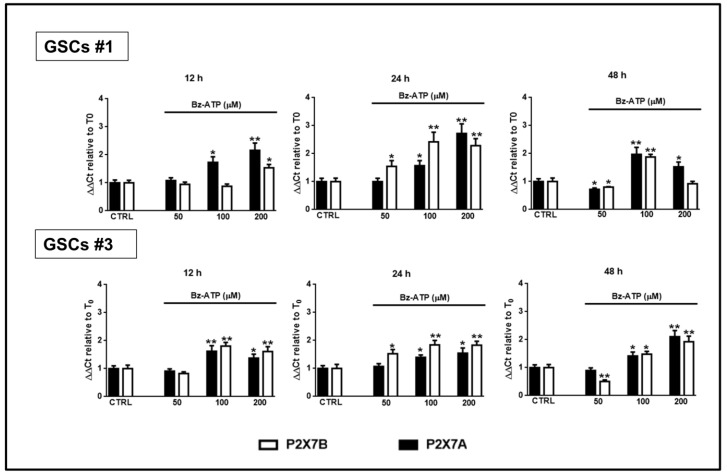
Modulation of the expression of the P2X7R splice variants A and B in GSCs exposed to BzATP. GSCs #1 and #3 were exposed to BzATP. At the indicated time points cells were collected to obtain their mRNA that was analyzed for the gene expression of the two splice variants P2X7R A and B. mRNA levels were normalized by using the house keeping β2-microglobulin as endogenous control. Values, calculated as fold of increase vs. untreated cells (CTR assumed as = 1), are the mean ± S.E.M. of three independent experiments in which each sample was tested in duplicate. *p < 0.05, **p < 0.01: statistical significance vs. untreated cells (one-way ANOVA plus Dunnett’s test).

Interestingly, cell exposure to BzATP induced an increased expression of mRNA for both splice variants, despite some differences. In GSCs #1, the expression of both isoforms was significantly increased even though that of the splice variant P2X7B was mainly evident at 24 h from drug treatment (Figure 12). 

**Figure 13:**
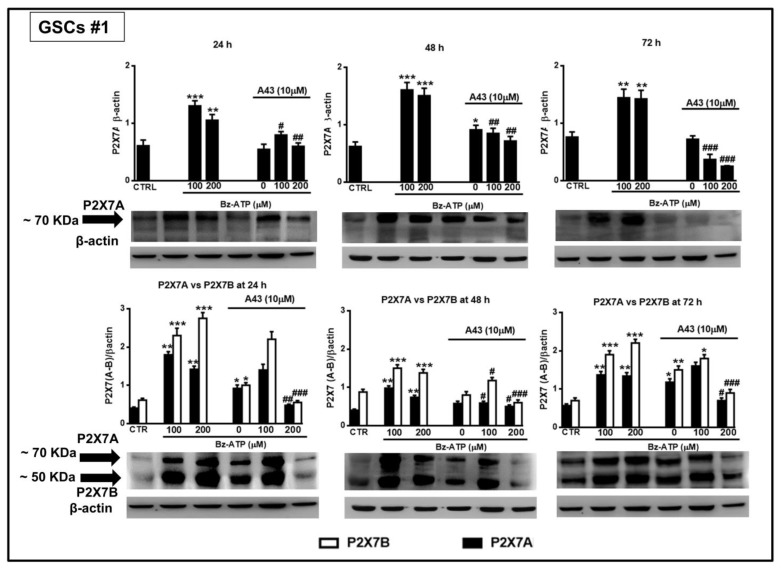
Modulation of the protein levels related to the P2X7R splice variants A and B in GSCs #1 exposed to BzATP. GSCs #1, cultured up to their confluence in vitro, were harvested at different time points after their exposure to BzATP and/or A438079. Protein levels of P2X7A and P2X7B isoforms were determined by western blot analysis. Immunoblots were obtained by exposing membranes to two antibodies, one recognizing only proteins at about 70 KDa and the second one recognizing proteins at about 70 and 50 KDa, respectively. Immunoblots were then re-probed with an antibody against β actin and quantified by densitometric analysis, the values of which, normalized to β actin used as an internal control, are reported in the histograms. The band of β-actin is the same for the blots at each time period, as the same membrane was first incubated with one antibody and then, after stripping, with the other antibody against the P2X7RA or B subunits. Densitometric values are the mean ± SEM of three independent experiments with very similar results. *p < 0.05, **p < 0.01, ***p < 0.001: statistical significance vs. untreated cells (CTR); #*p* < 0.05, ##*p* < 0.01, ###*p* < 0.001: statistical significance vs. cells treated with BzATP (one-way ANOVA plus Dunnett’s test).

**Figure 14:**
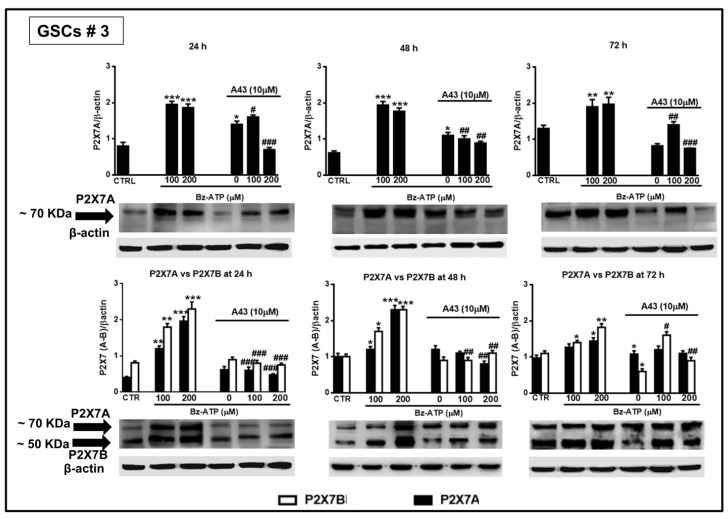
Modulation of the protein levels related to the P2X7R splice variants A and B in GSCs #3 exposed to BzATP. GSCs #3, cultured up to their confluence in vitro, were harvested at different time points following their exposure to BzATP and/or A438079. Protein levels of P2X7A and P2X7B splice variants were determined by western blot analysis as reported in the legend of the Figure 13. Immunoblots were re-probed with an antibody against β actin, quantified by densitometric analysis, normalized to β actin used as an internal control, and reported in the histograms. Densitometric values are the mean ± SEM of three independent experiments with very similar results. *p < 0.05, **p < 0.01, ***p < 0.001: statistical significance vs. untreated cells (CTR); #*p* < 0.05, ##*p* < 0.01, ###*p* < 0.001: statistical significance vs. cells treated with BzATP (one-way ANOVA plus Dunnett’s test).

In GSCs #3, the BzATP-induced up-regulation was similar for both P2X7R subunits along the entire observation period. However, these differences were nullified when the expression of the two splice variants was evaluated in terms of protein content. Indeed, BzATP showed a similar effect in GSCs #1 and # 3 up to 72 h (Figure 13 and Figure 14). 

Cell pretreatment with the P2X7R antagonist, A438079, counteracted the effects induced by BzATP on the two receptor subunits, especially when it was administered at 200 μM (Figure 13 and Figure 14).

## 4. Discussion

EMT is a complex process that, besides playing a crucial role during normal embryogenesis [38], has also been associated with the progression of many types of cancer [39]. As for GBM, it is also called epithelial-to-mesenchymal(-like) transition or glial-to-mesenchymal transition (GMT) [40,41], to underline that GBM is a non-epithelial derived tumor. However, a number of reports reported GMT as equivalent to EMT and showed its appearance as linked to GBM recurrence [7,42]. Since GSCs are regarded as cells triggering/supporting GBM malignancy, in this study we examined whether the modulation of EMT process in these cells might modify their aggressiveness. To note, we performed our experiments in the same cells used in a previous paper [24], derived from GBM of three different patients. Since these cells are not “cell lines”, we observed some differences in the onset and extent of drug effects, which, however, did not affect the overall validity of the results obtained, as discussed below.

EMT may be activated by different stimuli and, among them, we focused on purines, in particular ATP, being the environment around tumor cells, and in particular GBM, highly rich in ATP (few hundreds μmol/L) (reviewed by [23,24]). This is partially due to the low activity of enzymes metabolizing adenine nucleotides triphosphates in tumors compared to healthy tissues, as well as to the increased release of intracellular purines following cell death. Therefore, such high ATP concentration coupled to a longer permanence of the nucleotide at extracellular level is sufficient to activate P2X7R in glioma and, more in general, in brain tumors [43]. Thereby, it is not surprising that, using exogenous ATP at a relatively high concentration (300 μM) to stimulate GSC cultures, the expression levels of some key EMT markers related to GBM malignancy were enhanced, whereas cell pretreatment with a P2X7R antagonist reduced them. 

Based on these findings, we better investigated the effect of the activation of these receptors on the same EMT markers. Indeed, their expression increased from 12–24 up to 48–72 h following GSCs exposure to the P2X7R agonist, BzATP, as well as to TGFβ1, a well-known inducer of EMT in GBM [7,36]. However, mRNA analysis of the EMT markers showed some differences among the examined cells, which probably correlates with a different cell efficiency/rate of DNA transcription into mRNA in response to pharmacological stimulation. In contrast, the mRNA translation into the corresponding proteins resulted in a more constant response of the cells to the investigated drugs (TGFβ1 and BzATP). Thus, our data confirm the need to look also to the proteins codified by the corresponding mRNAs, which may more reliably account for drug effects. 

We also observed that both BzATP and TGFβ1 increased the GSC migration and invasion ability in vitro, which were tested after 24 h upon drug administration. Since mRNA or protein levels for most EMT markers analyzed were increased by the two drugs starting from the first 12–24 h, it is conceivable that also cell migration and invasion were significantly enhanced during the same period, even though data here not reported (personal observation) showed that the effect of the two drugs on cells invasion was evident even after 48 h following their administration. 

All these findings are consistent with literature, except data related to N-cadherin, which should not increase in GBM or, if increased, could restrain cell migration [17]. However, based on the controversy existing on the importance in the N-cadherin level change in GBM, the observed increase of this protein could be irrelevant for GSC invasiveness, which paralleled the general raise of other EMT markers induced by the pharmacological treatments reported here.

Collectively, our data demonstrate an involvement of P2X7R in directing GSCs towards EMT and enhancing/potentiating their aggressiveness, even though we cannot rule out the participation of other P2R. Indeed, GSC pre-treatment with A438079, a drug considered as one of the most selective P2X7R antagonists, completely counteracted or partially reduced the effect induced by 200 or 100 μM BzATP, respectively. These findings may be explained by the “partial” selectivity of BzATP towards P2X7R, being this agent able to stimulate also other ATP P2R at lower concentrations than those stimulating P2X7R [44]. Consequently, we cannot exclude that other members of the P2R family could contribute to increase EMT markers expression and GSC invasiveness. Given the growing roles potentially played by P2X7R not only in tumor development/growth, but also in many other physiological and pathological conditions, we believe as extremely important that selective agonists for P2X7R have to be delivered in commerce as soon as possible. 

We also explored a putative signaling pathway, which can be activated following P2X7R stimulation by BzATP. Based on the analogy of BzATP and TGFβ behavior in relation to EMT markers and GSC mobility/invasiveness, we checked whether they shared a common signal transduction pathway. Our results demonstrated that both agents, although to a different extent, increased the SMAD2 phosphorylation. Additionally, this effect was inhibited by cell pretreatment with an inhibitor of TGFβ type I receptors [36]. The same inhibitor also abrogated BzATP-induced increase in EMT markers and GSC invasion. Noteworthy, our results suggest that BzATP might favor the release of TGFβ from GSCs, which in turn would concur to increase their aggressiveness. Clearly, we cannot exclude the involvement of other molecular mechanisms more directly promoted by P2X7R activation, which need further investigation. 

Here, we demonstrated that, in GSCs, high expression of P2X7R is coupled to the presence of the two main splice variants in humans, which are the full length P2X7AR and the truncated isoform named P2X7BR, lacking the C-terminal 249 amino acids but provided with 18 extra amino acids after the residue 346. More importantly, the expression of both splice variants was increased in GSCs following their treatment with BzATP. While the receptor assembled with P2X7A monomers shows both ion channel and pore activity, the one deriving from P2X7B monomer assembly lacks pore function [27]. However, this may represent an over-simplification of the P2X7R molecular structure. Indeed, it has also been reported that the co-expression of P2X7A with P2X7B splice variants may result in the formation of a functional P2X7A-B heterotrimeric receptor that exhibits: i) An enhanced pore activity or ii) a higher affinity for ATP than a homotrimeric P2X7R and an increased support to cell energy and metabolism [25]. These conflicting results can be reconciled by a unifying explanation about the effects consequent to homo- or hetero-trimeric P2X7R activation, according to which the overstimulation of these receptors by high micromolar or millimolar ATP concentrations causes cell death, whereas tonic P2X7R stimulation at lower ATP concentrations favors cell survival/growth [21,23]. Consistently with this explanation, we previously found that ATP or BzATP administered at high concentrations and for two consecutive days caused GSC death. In contrast, in this paper we revealed that BzATP, administered only once at lower concentrations (100 and 200 μM), protected GSCs from apoptosis and did not induce necrosis. Thereby, it is not surprising that the P2X7R antagonist, when administered alone to the cells, increased LDH and caspase activity whereas, when co-administered with BzATP, reduced the protective effects of this agent against necrotic or apoptotic GSC death. On the other hand, the decrease in cell viability reported at 96 h after cell exposure to the highest BzATP concentration (200 μM) should not be due to cell death, but rather to an arrest in S phase of the cell cycle, as we previously demonstrated for the same cells following exposure with higher BzATP concentration (500 μM). Finally, the up-regulated expression of both P2X7R A and B variants induced by BzATP might also favor the increase in EMT marker expression and GSC invasiveness, provided that relatively low P2X7R agonist concentrations are used. 

In conclusion, the effects induced in human GSCs by a “moderate” P2X7R stimulation could enhance GBM aggressiveness, even though the occurrence of these effects needs to be verified in vivo. If ascertained, it would follow that the GBM growth could be restrained by inhibiting P2X7R, as observed in other tumors [45]. Clearly, we are aware that GBM progression depends on multiple factors and that the expression of P2X7R might be different in tumor core or periphery, as observed for pancreatic tumor [46], or present also in peri-tumor cells, such as resident microglia or infiltrating macrophages, which may support tumor growth by releasing P2X7R-dependent factors [47]. Thus, further research needs to define this complex scenario and the role of P2X7R therein, in order to advantageously apply the pharmacological modulation of P2X7R expression/activity in GBM therapeutic management. 

## Abbreviations

A438079, 3-[[5-[2,3-Dichlorophenyl]-1*H*-tetrazol-1-yl]methyl]pyridine hydrochloride;
A8301, 3-[6-methyl-2-pyridinyl]-*N*-phenyl-4-[4-quinolinyl]-1*H*-pyrazole-1-carbothioamide;
Bz-ATP, 2′[3′]-*O*-[4-benzoylbenzoyl]-ATP;
EMT, epithelial-to-mesenchymal transition;
GBM, glioblastoma multiforme;
GMT, glial-to-mesenchymal transition;
GSCs, glioblastoma-derived stem cells;
MTS, 3-[4,5-dimethylthiazol-2-yl]-5-[3-carboxymethoxy phenyl]-2-[4-sulfophenyl]-2*H*-tetrazolium; P2R, P2 receptors;
P2X7R, P2X7 receptors;
P2X7AR and P2X7BR, P2X7A and P2X7B receptors;
qRT-PCR, quantitative real time polymerase chain reaction;
Snail1, snail family transcriptional repressor 1;
TGFβ1, transforming growth factor β1;
ZEB1, zinc finger E-box binding homeobox1.
